# Risk factors for major adverse cardiovascular events after the first acute coronary syndrome

**DOI:** 10.1080/07853890.2021.1924395

**Published:** 2021-06-03

**Authors:** Marjo Okkonen, Aki S. Havulinna, Olavi Ukkola, Heikki Huikuri, Arto Pietilä, Heli Koukkunen, Seppo Lehto, Juha Mustonen, Matti Ketonen, Juhani Airaksinen, Y. Antero Kesäniemi, Veikko Salomaa

**Affiliations:** a Research Unit of Internal Medicine, University of Oulu, Oulu, Finland; b Medical Research Center Oulu, Oulu University Hospital, Oulu, Finland; c Finnish Institute for Health and Welfare, Helsinki, Finland; d FIMM: Institute for Molecular Medicine Finland, Helsinki, Finland; e Kuopio University Hospital, Kuopio, Finland; f University of Eastern Finland, Kuopio, Finland; g North Karelia Central Hospital, Joensuu, Finland; h University of Turku and Heart Center Turku University Hospital, Turku, Finland

**Keywords:** Epidemiology, acute coronary syndromes, prevention, major adverse cardiac event

## Abstract

**Aims:**

To evaluate risk factors for major adverse cardiac event (MACE) after the first acute coronary syndrome (ACS) and to examine the prevalence of risk factors in post-ACS patients.

**Methods:**

We used Finnish population-based myocardial infarction register, FINAMI, data from years 1993–2011 to identify survivors of first ACS (*n* = 12686), who were then followed up for recurrent events and all-cause mortality for three years. Finnish FINRISK risk factor surveys were used to determine the prevalence of risk factors (smoking, hyperlipidaemia, diabetes and blood pressure) in post-ACS patients (*n* = 199).

**Results:**

Of the first ACS survivors, 48.4% had MACE within three years of their primary event, 17.0% were fatal. Diabetes (p = 4.4 × 10^−7^), heart failure (HF) during the first ACS attack hospitalization (p = 6.8 × 10^−15^), higher Charlson index (p = 1.56 × 10^−19^) and older age (*p* = .026) were associated with elevated risk for MACE in the three-year follow-up, and revascularization (*p* = .0036) was associated with reduced risk. Risk factor analyses showed that 23% of ACS survivors continued smoking and cholesterol levels were still high (>5mmol/l) in 24% although 86% of the patients were taking lipid lowering medication.

**Conclusion:**

Diabetes, higher Charlson index and HF are the most important risk factors of MACE after the first ACS. Cardiovascular risk factor levels were still high among survivors of first ACS.

## Introduction

Cardiovascular diseases (CVD) are the leading cause of death in many countries. In 2017 17.8 million people died because of a CVD event [1]. Many studies have been published on risk factors for coronary heart disease (CHD) which is the leading component of CVD [1,2]. Age, male sex, hypertension, hyperlipidaemia, diabetes and cigarette smoking are commonly considered as risk factors for CHD [3–6]. Also, left ventricular systolic dysfunction, severity of CHD and comorbidities are associated with high risk of an adverse outcome [3,5,7].

Different risk indicators for major adverse cardiac event (MACE) after an acute coronary syndrome (ACS), such as angiographic outcome and left ventricular function, have been previously observed [6,7]. Also, the short-term risk of MACE after ACS is well known [5,8,9]. However, the long-term risk factors for MACE after an ACS have been rarely examined [5,8,10,11]. Previous studies have focussed on MACE after either a non-ST-elevation myocardial infarction (NSTEMI) or ST-elevation myocardial cinfarction (STEMI) and current studies rarely separate between MACE after the first event or recurrent event [5,7,11,12].

Major improvements have taken place in diagnosis and treatment of ACS during past decades [13]. This may have led to changes in most common risk factors for MACE after ACS. The risk factors may be different for first versus recurrent event and the risk factors for long- and short-term recurrence may differ. Therefore, it is important to evaluate the entire spectrum of MACE after ACS in a long-term study.

The purpose of our study was to evaluate risk factors for MACE within one- and three-yeartime periods after the first ACS event. In particular we wanted to identify modifiable risk factors, which could provide treatment targets for further improvements in prognosis after the first ACS. We also examined the prevalence of risk factors (smoking, hyperlipidaemia, diabetes and blood pressure) among a population-based sample of post-ACS patients from the FINRISK risk factor survey-populations to gain understanding of the current treatment and control levels of these risk factors.

## Methods

This study was based on a follow-up of the FINAMI participants. The FINAMI register has been described previously [14]. In brief, it was a population- based myocardial infarction register, which was functioning in four different geographical areas of Finland. FINAMI registered all events from Oulu, Joensuu, Turku and Kuopio areas that were or were suspected to be myocardial infarction (MI), unstable angina pectoris (UAP) or CHD death [14]. Trained nurses collected the data manually from hospital records [14]. FINAMI has received approval from the Ethics Committee of the National Institute for Health and Welfare during 21 January 2004.

Diagnostic classification of events was done according to the American Heart Association (AHA) Scientific Statement of 2003 [15] and before that according to the WHO MONICA Project criteria [16]. The duration of attack was defined as 28 days after onset of the attack. The most severe symptoms and findings during this time frame were recorded [14]. The attack was defined as the first if there was no mention of previous symptomatic events in the hospital records.

All symptomatic patients registered for their first ACS in1993–2011 aged 35 to 74 years were included in this study. Included patients did not have any previous symptomatic events according to the hospital records and they had survived the first 28 days of their first ACS event. MACE during the follow-up was identified by record linkage of the FINAMI data based on the personal ID code with the National Hospital Discharge Register (NHDR) and the Causes of Death Register (CDR). Endpoints of the follow up (MACE) were new fatal or non-fatal ACS (UAP or MI), fatal or non-fatal heart failure (HF), fatal or non-fatal stroke, fatal or non-fatal CVD event and all-cause death. These events were identified from the NHDR and the CDR using international classification of diseases (ICD) 9 and 10 (Finnish version) codes. Please see appendices for the specific codes.

We also examined a sample of FINRISK survey [17] participants from survey years 2007 (*n* = 6258) and 2012 (*N* = 5827) to assess the prevalence of identified cardiovascular risk factors amongst those participants who had previously had ACS. FINRISK population has been described in more detail in our previous article [14]. In short, these patients were from the same geographic areas as the FINAMI patients and were aged 25–74 years. The same ICD 10 (Finnish version) codes were used to identify patients with a history of ACS event as for the FINAMI population.

### Statistical methods

Examination of the data revealed that the proportional hazards assumption was not fulfilled in time to event models and therefore logistic regression was used to calculate the odds ratios and confidence intervals for risk factors of MACE. The models were adjusted for age, sex (when relevant), study area and the year of the event. Comparison of risk factors for MACE between patients with different ACS event-types was done using multivariate analysis which was adjusted for age, study area and the year of event. Charlson index [18] was used to evaluate the effect of comorbidity on mortality. It gives weighted points to patients comorbidities [18]. History of MI, HF, peripheral vascular disease, cerebrovascular disease, para- and hemiplegia, chronic obstructive lung disease, rheumatoid disease, peptic ulcer disease, mild liver disease and diabetes mellitus (DM) are weighed with one point. DM with target organ damage, dementia, renal disease and any cancer were weighted two points. Moderate to severe liver disease was weighted with three points and metastatic solid tumour and AIDS (stage C) were weighted with six points [18]. For the FINRISK population, the prevalence of current smoking, high blood cholesterol level, use of lipid lowering drugs, diabetes and hypertension were estimated.

## Results

Altogether, 12,686 patients survived 28 days after their first ACS episode (Table 1). This was 61.9% of the total first ever ACS patient population. Of these patients, 34.4% had MACE within one year and 48.4% within three years of their primary event. Of these recurrent MACE events, 17.6% were fatal within three years. We also carried out a separate analysis, including only the latest study years, 2006 − 2011. During this period, altogether 3261 patients survived 28 days after their first ACS episode. This was 62.3% of the total first ever ACS patient population. Of these patients, 36.2% had MACE within one year and 48.4% within three years of their primary event. Of these recurrent MACE events, 15.5% were fatal within three years. Among the whole study population, the average age of having MACE after the first ACS was 67.5 years for men and 76.9 for women. The average time from ACS to MACE was less than one year (0.77 years).

**Table 1.** Clinical characteristics of the study population.

	Men	Women	Total
Survivors after first ACS (%*)	6676 (62.8)	6010 (60.9)	12,686 (61.9)
Death within 1 year (%)	661 (9.9)	881 (14.7)	1542 (12.2)
Death within 3 years (%)	1369 (20.5)	1783 (29.7)	3152 (24.9)
MACE within 1 year (%**)	2221 (33.3)	2143 (35.7)	4364 (34.4)
MACE within 3 years (%)	3019 (45.2)	3075 (51.2)	6094 (48.4)
Fatal MACE within 3 years (%)	852 (12.8)	1300 (21.6)	2152 (17.0)
Average age at MACE (SD)	67.5 (11.9)	76.9 (10.9)	72.0 (12.3)
Time from the first ACS to subsequent MACE in years (SD)	0.73 (0.76)	0.80 (0.78)	0.77 (0.77)
Stroke within 3 years (%)	448 (6.7)	522 (8.7)	970 (7.7)
ACS within 3 years (%)	2281 (34.2)	2092 (34.8)	4373 (34.5)
HF within first 28 days (%)	817 (12.2)	1231 (20.5)	2048 (16.4)
Prevalent DM among 28-day survivors (%)	729 (10.9)	872 (14.5)	1601 (12.6)
Current- / ex- / non-smokers among 28-day survivors (%)	1371 / 1290 / 721 (40.5 / 38.1 / 21.3)	480 / 314 / 1520 (20.7/ 13.6 / 65.7)	1851 / 1604 / 2241 (32.5 / 28.2 / 39.3)
Total cholesterol, mean (SD) N (%) of cholestero*l* > 5mmol/l	4.87 (1.18) 2103 (41.7)	5.05 (1.27) 1608 (47.2)	4.94 (1.22) 3711 (43.9)

ACS: acute coronary syndrome; MACE: major adverse cardiac event; SD: standard deviation; MI: myocardial infarction; HF: heart failure; DM: diabetes mellitus.

*Percent of all first ACS cases.

** Percent of first MI survivors.

DM and HF within the first 28 days of first ACS attack and higher Charlson index were associated with higher risk of MACE at one-year follow-up (Table 2). In the three-year follow-up higher age and the same risk factors: DM, HF and Charlson index were associated with higher risk of MACE. Only revascularization was a protective factor in both one- and the three-year follow-up.

**Table 2.** Risk factors for MACE after the first ACS event. All the risk factors were modelled independently of other risk factors and adjusted for age, sex and event year.

	MACE at one year (*n* = 4364) OR (CI / *p* Value)	MACE at three years (*n* = 6094) OR (CI / *p* Value)
Age (per 10 years)	0.94 (0.88–1.01/.08)	1.07 (1.01–1.14/.026)
Sex (=female)	0.88 (0.77–1.02/.095)	0.93 (0.81–1.06/.28)
Event year	0.99 (0.97–1.00/.13)	0.98 (0.97–1.00/.077)
Diabetes	1.39 (1.17–1.64/1.3 × 10^−4^)	1.51 (1.29–1.77/4.4 × 10^−7^)
Cholesterol (per SD)	1.00 (0.92–1.07/.93)	0.96 (0.9–1.03/.28)
Current smoking	1.12 (0.96–1.31/.14)	1.14 (0.98–1.31/.081)
Revascularization (within 28 days)	0.84 (0.73–0.97/.021)	0.82 (0.71–0.94/.0036)
Primary HF	1.95 (1.63–2.34/1.9 × 10^−13^)	1.98 (1.67–2.36/6.8 × 10^−15^)
Primary AF	1.1 (0.91–1.33 /.33)	1.11 (0.93–1.33/.25)
Use of thrombolytic treatment	1.12 (0.94–1.32/.2)	1.18 (1.01–1.38/.035)
Charlson index* per 1 point	1.11 (1.09–1.13/1.94 × 10^−21^)	1.09 (1.07–1.12/1.56 × 10^−19^)
UAP (as AHADG, 15.4% of all cases)	0.99 (0.83–1.17/0.89)	1.11 (0.95–1.3/.18)

MACE: major adverse cardiac event; ACS: acute coronary event; OR: odds ratio; CI: confidence interval; SD: standard deviation; HF: heart failure; AF: atrial fibrillation.

*Calculated in separate model, because the Charlson index includes primary HF and AF, adjusted for age and sex. The other ORs were produced in the same model.

Age, DM, and Charlson index were associated with higher risk of stroke both in one- and three-year follow-up (Table 3). Female sex and revascularization were protective from stroke at one-year time point. At three-year follow-up higher blood cholesterol was inversely associated with stroke risk.

**Table 3.** Risk factors for stroke after the first ACS event. All the risk factors were modelled independently of other risk factors and adjusted for age, sex and event year.

	Stroke at one year (*n* = 545 (me*n* = 250 and wome*n* = 295)) OR (CI / *p* Value)	Stroke at three years (*n* = 970 (me*n* = 448 and wome*n* = 522)) OR (CI / *p* Value)
Age (per 10 years)	1.37 (1.14–1.65 / 9.3 × 10^−4^)	1.41 (1.23–1.63/ 1.4 × 10^−6^)
Sex (=female)	0.66 (0.45–0.97/.033)	0.79 (0.59–1.05/.10)
Event year	1.00 (0.95–1.06/.85)	0.98 (0.94 − 1.01/.22)
Diabetes mellitus	1.84 (1.24–2.72/ .0022)	1.82 (1.35–2.43/ 6.6 × 10^−5^)
Cholesterol (per SD)	0.9 (0.73–1.1/.31)	0.82 (0.7–0.96/.014)
Current Smoking	0.96 (0.63-1.48/.87)	0.92 (0.67–1.28/.64)
Revascularization (within 28 days)	0.63 (0.42–0.93/ .021)	0.83 (0.62–1.12/.22)
Primary HF	0.69 (0.42–1.12/ .13)	0.8 (0.56-1.13/.21)
Primary AF	0.97 (0.6–1.57/.92)	0.98 (0.69-1.39/.9)
Use of thrombolytic treatment	0.82 (0.49-1.37 /.44)	1.06 (0.75-1.50 /.74)
Charlson index* per 1 point	1.22 (1.10–1.36/ 2.9 × 10^−4^)	1.14 (1.05–1.25 / .0026)
UAP as index event (15.4% of all cases)	1.18 (0.76–1.84/.45)	0.95 (0.67–1.35 / .78)

ACS: acute coronary event; OR: odds ratio; CI: confidence interval; SD: standard deviation; HF: heart failure; AF: atrial fibrillation.

*Calculated in separate model, because the Charlson index includes primary HF and AF, adjusted for age and sex. The other ORs were produced in the same model.

Table 4 shows risk factors for fatal MACE within three years of index event (*n* = 2152). Older age, diabetes and primary HF increased the risk of fatal MACE and protective factors were revascularization and later year of the event.

**Table 4.** Risk factors for fatal MACE (*n* = 2152) within three years of index event.

	OR	CI	*p* Value
Event Year	0.92	(0.9–0.95)	7.9e-8
Sex (female)	0.83	(0.68–1)	.053
Age per 10 years	1.53	(1.39–1.7)	4.6e-17
Diabetes	2.38	(1.94–2.91)	7.3e-17
Cholestero*l* > 5 vs. ≤5	0.95	(0.86–1.05)	.3
Current smoking	1.13	(0.9-1.42)	.28
Revascularization (within 28 days)	0.56	(0.45–0.7)	1.5e-7
Primary HF	2.46	(1.99–3.02)	3e-17
Primary AF	1.02	(0.81–1.28)	.89
UAP as index event (15.4% of all cases)	1.04	(0.82–1.32)	.75

MACE: major adverse cardiac event; OR: odds ratio; CI: confidence interval; HF: heart failure; AF: atrial fibrillation; UAP: unstable angina pectoris.

The prevalence of risk factors among FINRISK participants (from survey-years 2007 and 2012) who had a history of their first ACS (*N* = 199) is shown in Table 5. After the ACS event, 23% of patients were still smoking. The number of patients currently taking lipid lowering drugs after ACS event was high (86%) but still 24% of them had total cholesterol 5 mmol/l or more.

**Table 5.** Prevalence of known risk factors among FINRISK 2007 and 2012 survey participants with a history of first ACS event.

Variable	Men	Women	Total
*N*	158 (100%)	41 (100%)	199 (100%)
Smoking	34 (21%)	11 (27%)	45 (23%)
Cholesterol (>5mmol/l)	27 (17%)	21 (54%)	48 (24%)
Lipid lowering drug use	144 (91%)	27 (66%)	171 (86%)
Diabetes mellitus	57 (36%)	16 (39%)	73 (37%)
Hypertension (Bp >140/90 or on medication)	114 (74%)	29 (74%)	143 (74%)

ACS: acute coronary syndrome; BP: blood pressure.

## Discussion

The risk of recurrent MACE was high among survivors of first ever ACS and a substantial proportion of these events was fatal. Of first ever ACS patients 34.4% had MACE within one year and 48.4% within three years of their primary event. Previous studies have shown that 20.4% to 36.7% patients have MACE after ACS. The proportion is lower for patient who have had revascularization during the first attack [12,19]. DM and HF during the first attack and high Charlson comorbidity index were associated with a higher risk for MACE within one- and three- years after the first ACS event. Higher age was associated with higher risk in the three-year follow-up. A revascularization procedure was the only protective factor in both one- and three-year time points. In FINRISK population, the risk factors were still sub-optimally controlled amongst patients who had survived one ACS event.

Not surprisingly, the higher Charlson comorbidity index was associated with a higher risk for MACE and stroke within one and three years after the first ACS event in our study. It is likely that this is mainly due to DM and HF being part of Charlson index. However, other risk factors may also play a role since a previous study by Radovanovic et al. [20] shows higher prevalence of elevated blood cholesterol, hypertension and obesity in people with higher Charlson index.

Major improvements have taken place in the diagnosis and treatment of ACS during our study period [13,14]. In our study population from the years 1993–2011, only one in four had angiographies during the first attack. Revascularization became better available in Finland only after the year 2000 and has now reached a plateau [21]. Even though the number of patients who had angiography during first attack period was relatively small in our study, revascularization was still protective factor. This is in line with previous studies [5,19,22]. To explore the effects of improved treatments, we carried out a separate analysis for the later study years, 2006–2011. The results showed an improvement in both short- and long-term survival, but among survivors the proportion of recurrent MACE events remained the same. Because of the amount of revascularization procedures has reached a plateau [21] it is fair to assume that if we aim to improve long-term prognosis of ACS patients we should also target the modifiable risk factor.

Most important modifiable risk factors for CVD include hypertension, smoking, DM, obesity, physical inactivity, unhealthy diet, high cholesterol and other lipids and psychosocial factors [23–24]. In our study, cholesterol was not a statistically significant risk factor for MACE after ACS, which may be due to widespread use of statins after the first ACS event. In our FINRISK population 86% of patients were taking lipid lowering medication. However, 24% of the patients still had total cholesterol 5 mmol/l or more even with lipid lowering medication.

Smoking at the time of the first ACS tended to be associated with elevated risk of future MACE but did not reach statistical significance probably because many patients stop smoking after the first ACS. However, in our FINRISK sample 23% of patients were still smoking after their first ACS. The FINRISK population showed suboptimal secondary prevention after the first ACS. Our sample size of post-ACS patients is smallish, but the findings are consistent with the EUROASPIRE study [25].

A previous study by our group [26] showed that regular use of secondary preventive medications lowers the risk of recurrent ACS after the primary attack and that the use of medications was suboptimal in the elderly and in patients with diabetes. In our present study the elderly and patients with DM were both at higher risk of MACE after ACS and the suboptimal use of medication, and thus suboptimal secondary prevention, could in part explain the high MACE rate for these patients. The specific medication data was not available for our study and we did not have the blood pressure values either.

The strength of our study is the FINAMI register which was a large, population-based register covering all ACS cases in monitored populations. FINAMI data enabled us to identify and analyse all first ACS events in populations of the register areas, and the record linkage of FINAMI data to the NHDR and the CDR through the personal ID number made it possible to identify patients who had MACE after the first ACS event. This ensured complete identification of the MACE patients who were hospitalized or who died either in hospital or out of hospital.

In addition to the previously mentioned limitations, FINAMI does not separate NSTEMI from STEMI. Therefore, we could not consider different types of MI. While NHDR diagnosis for MI has shown to be valid [27] this is not the case for the NSTEMI and STEMI diagnoses [28].

In conclusion, DM and HF are the most important risk factors for MACE after ACS. Revascularization was clearly protective factor. Also, the treatment of risk factors seems to be at suboptimal level among the survivors of first ACS. Based on our findings in FINRISK population, especially hypercholesterolaemia should be treated more aggressively and patients should be actively encouraged to stop smoking. This highlights the importance of secondary prevention for ACS patients and also the significance of up-to-date scientific research on risk factors and their changes in populations.

## APPENDIX

ICD codes

International Classification of Diseases (ICD) codes were used to cross-check the FINAMI data with the National Hospital Discharge Register (NHDR) and the Causes of Death Register (CDR). ICD 9 (Finnish version) was used until the end of 1995 and ICD 10 (Finnish version) after that. Out of hospital deaths were evaluated if the underlying or direct cause of death was coronary heart disease (ICD 9: 410–414, ICD 10: I20–I25) or cardiac arrest (I46.1, I46.9). Sudden death was also considered for registration if the cause was unknown or unattended death (ICD 9: 798, ICD 10: R96, R98). The National Hospital Discharge register was cross-checked for hospitalisation due to MI or UAP (ICD 9: 410–411, ICD 10: I20–22). The local registration teams evaluated these cases according to the registration protocol [1,2]. NHDR and CDR have been validated earlier [3]. Stroke was identified using ICD 9 codes 431, 4330 A, 4331 A, 4339 A, 4340 A, 4341 A, 4349 A and 436, and ICD 10 codes I61, I63 and I64 (except I63.6 was excluded) from the NHDR and the CDR. Heart failure was identified using ICD 9 codes 428and 4289X, and ICD 10 codes I50 and I50.0 from the NHDR and CDR.
